#  As informações sobre os direitos sociais estão acessíveis aos pacientes oncológicos? 

**DOI:** 10.1590/0102-311XPT096023

**Published:** 2023-09-25

**Authors:** Fernanda Guedim Batista, André Salem Szklo

**Affiliations:** 1 Associação Feminina de Prevenção e Combate ao Câncer, Juiz de Fora, Brasil.; 2 Instituto Nacional de Câncer José Alencar Gomes da Silva, Rio de Janeiro, Brasil.

**Keywords:** Direitos Socioeconômicos, Processo Saúde-Doença, Legislação Médica, Sistema Único de Saúde, Socioeconomic Rights, Health-Disease Process, Medical Legislation, Unified Health System, Derechos Socioeconómicos, Proceso Salud-Enfermedad, Legislación Médica, Sistema Único de Salud

## Abstract

A legislação brasileira assegura aos pacientes com câncer direitos que auxiliam no tratamento e atenuam os gastos despendidos na jornada de adoecimento. O objetivo do estudo foi calcular a proporção de indivíduos em tratamento oncológico de um centro de referência do Sistema Único de Saúde (SUS) que referiram conhecer 15 direitos específicos previstos em lei, segundo o subgrupo populacional elegível para solicitar cada direito. Foram entrevistados todos os pacientes oncológicos adultos em início de tratamento no Hospital Associação Feminina de Prevenção e Combate ao Câncer de Juiz de Fora (ASCOMCER), Minas Gerais, entre março e julho de 2022 (n = 62). Cerca de 60% desses pacientes eram analfabetos ou não tinham completado o ensino fundamental, aproximadamente 75% viviam em domicílios em que a renda *per capita* era de no máximo um salário mínimo e 91,9% eram atendidos pelo SUS. Para nove dos 15 direitos selecionados, a proporção de pacientes elegíveis foi superior a 10%, variando de 17,7% para “saque do Fundo de Garantia por Tempo de Serviço (FGTS)” a 100% para “prioridade na tramitação de processos”. No entanto, o único desses direitos conhecido por pelo menos 50% dos pacientes elegíveis foi o “auxílio-doença” (70,6%), sendo que para três direitos as respectivas proporções não chegaram a 5% (isenção de imposto sobre propriedade predial e territorial urbana, isenção do imposto de renda na aposentadoria, pensão e reforma e prioridade na tramitação de processos). Os pacientes oncológicos necessitam ter seus cuidados integrais fortalecidos. Dessa forma, é fundamental aumentar o acesso à informação sobre os benefícios que eles podem obter de um Estado democrático de direito.

## Abstract (en)

Brazilian legislation provides for rights for cancer patients in order to assist with their treatment and mitigate the expenses they face during their illness. This study aimed to calculate the proportion of individuals undergoing cancer treatment at a Brazilian Unified National Health System (SUS) referral center who reported being aware of 15 specific legal rights, according to the population subgroup eligible to request each right. All adult cancer patients starting treatment at the Juiz de Fora Women’s Association for Preventing and Fighting Cancer Hospital (ASCOMCER), Minas Gerais State, from March to July 2022, were interviewed (n = 62). About 60% of these patients had incomplete primary education or were illiterate, around 75% lived in households with a per capita income below one minimum wage, and 91.9% received treatment from the SUS. For nine of the 15 selected rights, the proportion of eligible patients was higher than 10%, ranging from 17.7% for “Withdrawal from the Severance Pay Fund (FGTS)” to 100% for “priority in the processing of procedures”. However, the only one of these rights known to at least 50% of eligible patients was “sickness benefit” (70.6%). The respective proportions were below 5% in three rights, including “exemption from property tax”, “exemption from income tax on retirement, pension, and retirement”, and “priority in the processing of cases”. Cancer patients need to have their comprehensive care strengthened. Therefore, it is crucial to enhance the availability of information regarding the benefits cancer patients may receive from a democratic state that respects the rule of law.

## Abstract (es)

La legislación brasileña garantiza a los pacientes con cáncer derechos que les ayudan en el tratamiento y mitigan los gastos que tienen a lo largo de la enfermedad. El objetivo de este estudio fue calcular la proporción de individuos sometidos al tratamiento oncológico en un centro de referencia del Sistema Único de Salud (SUS) brasileño que informaron conocer 15 derechos específicos previstos por la ley, según el subgrupo de población elegible para solicitar cada derecho. Se entrevistó a todos los pacientes adultos con cáncer que comenzaron el tratamiento en el Hospital Asociación de Mujeres para la Prevención y Lucha contra el Cáncer de Juiz de Fora (ASCOMCER), Minas Gerais, entre marzo y julio de 2022 (n = 62). Cerca del 60% de estos pacientes eran analfabetos o no habían completado la escuela primaria, aproximadamente el 75% vivía en hogares con ingreso per cápita de un salario mínimo y el 91,9% eran tratados por el SUS. Para 9 de los 15 derechos seleccionados, la proporción de pacientes elegibles para ellos fue superior al 10%, oscilando del 17,7% para el “retirada del Fondo de Garantía por Duración del Servicio” al 100% para “prioridad en la tramitación de procedimientos”. Sin embargo, de estos derechos el único conociodo por al menos el 50% de los pacientes elegibles conocía fue el “beneficio por enfermedad” (70,6%), y en 3 derechos las proporciones respectivas no alcanzaron ni siquiera el 5% (“exención del impuesto sobre bienes inmuebles y territorial urbano”, “exención del impuesto sobre la renta por jubilación, pensión y retiro” y “prioridad en la tramitación de procedimientos”). Los pacientes con cáncer requieren el fortalecimiento de una atención integral. Por lo tanto, es esencial incrementar el acceso a la información sobre los beneficios que los pacientes con cáncer pueden obtener de un Estado de derecho democrático.

## Introdução

O câncer é um problema mundial cuja incidência e mortalidade vêm aumentando, estando entre as quatro principais causas de morte precoce na maioria dos países. No Brasil, estimam-se, para o triênio 2023-2025, 704 mil novos casos de câncer por ano ^1^.

Nos países em desenvolvimento, observa-se uma transição na incidência dos tipos de câncer, com queda daqueles predominantemente associados a infecções e aumento daqueles relacionados à melhoria das condições socioeconômicas e à incorporação de hábitos e atitudes relativos à urbanização, tais como sedentarismo e comportamentos alimentares inadequados ^2^. É fundamental, portanto, estar preparado para o crescimento dessas enfermidades nos sistemas de saúde, adotando medidas de prevenção, diagnóstico precoce, distribuição de recursos para tratamento apropriado, tendo em vista um aumento na complexidade e nos custos anuais ^1^^,^^2^.

Especificamente no Brasil, o Sistema Único de Saúde (SUS) proporcionou, com sua regulamentação em 1990, o acesso universal, sem discriminação, ao sistema público de saúde ^3^. A atenção integral à saúde, e não somente aos cuidados assistenciais, passou a ser um direito de todos os brasileiros, com foco na saúde com qualidade de vida, visando à prevenção e à promoção da saúde. Posteriormente, foram instituídas e aprimoradas políticas públicas de prevenção e controle do câncer no sentido de reforçar a atribuição ao Estado do dever de desenvolver políticas de saúde específicas voltadas à pessoa com câncer ^4^^,^^5^. Nesse contexto, e considerando que durante o tratamento o paciente pode ter gastos extras que prejudicam a dinâmica familiar, vale a pena destacar que a legislação brasileira assegura aos pacientes portadores de doenças graves e câncer, bem como a seus familiares, direitos que auxiliam no tratamento e, especificamente, atenuam as despesas que são necessárias nesse período de adoecimento (Quadro 1) ^6^^,^^7^^,^^8^.

**Quadro 1:** Direitos assegurados pela legislação brasileira a pacientes portadores de doenças graves e câncer, bem como a seus familiares, que auxiliam no tratamento e atenuam as despesas que são necessárias no período de adoecimento.

DIREITO	DESCRIÇÃO
Benefício de Prestação Continuada da Assistência Social (BPC)	É um benefício instituído pela Lei Orgânica de Assistência Social (LOAS) e integra a Proteção Social Básica no âmbito do Sistema Único de Assistência Social (SUAS). É um direito do deficiente ou da pessoa idosa (65 anos ou mais) que comprove não ter meios de prover seu sustento e de sua família. Para ter direito a esse benefício, a renda *per capita* familiar deve ser inferior a 1/4 do salário mínimo. O paciente oncológico passará por uma avaliação financeira e por uma avaliação médica realizada pelo perito do Instituto Nacional do Seguro Social (INSS).
Auxílio-doença	É um benefício garantido quando o segurado está temporariamente incapaz de exercer suas atividades laborativas. Tem direito a esse benefício pacientes com câncer que sejam segurados do INSS.
Aposentadoria por invalidez	É um benefício garantido ao trabalhador, segurado do INSS, que estiver impossibilitado definitivamente de exercer suas atividades laborativas. É concedido a partir da solicitação de auxílio-doença. A pessoa com câncer terá direito ao benefício desde que esteja na qualidade de segurado.
Isenção de Imposto sobre Produtos Industrializados (IPI) e Imposto sobre Operações financeiras (IOF)	É uma taxa federal que incide também no financiamento de automóveis. O paciente oncológico poderá ser isento desse imposto apenas quando apresentar deficiência física, visual, mental severa ou profunda. Pacientes com câncer que ficaram com alguma sequela em membros superiores e inferiores podem requerer a isenção.
Isenção de Imposto sobre a Propriedade de Veículos Automotores (IPVA) para veículos adaptados	É o imposto sobre propriedade de veículos automotores pagos anualmente pelo proprietário do carro. A legislação pode variar em cada estado, mas a maioria isenta o deficiente físico de pagar o IPVA sobre veículos de fabricação nacional, assim como os pacientes oncológicos que ficaram com alguma sequela em membros superiores e inferiores.
Isenção de Imposto sobre Circulação de Mercadorias e Serviços (ICMS)	É o imposto estadual sobre operações relativas à circulação de mercadorias e sobre prestação de serviços. Cada estado tem legislação própria. Geralmente, só têm direito os pacientes com câncer que ficaram com alguma sequela em membros superiores ou inferiores.
Quitação de financiamento de imóvel pelo Sistema Financeiro de Habitação (SFH) em caso de invalidez ou morte	Têm direito à quitação, caso haja cláusula no contrato, pessoas com invalidez total ou permanente, causada por acidente ou doença. É necessário que estejam inaptas ao trabalho e o contrato de compra do imóvel deverá ter sido assinado antes da incapacidade.
Saque do Fundo de Garantia por Tempo de Serviço (FGTS)	FGTS é a soma de depósitos mensais que a empresa em que o indivíduo trabalha é obrigada a fazer em seu nome. Todos os trabalhadores com câncer que têm carteira assinada, estão registrados em regime da Consolidação das Leis do Trabalho (CLT) e têm uma conta bancária vinculada ao seu contrato de trabalho administrada pela Caixa Econômica Federal têm direito ao benefício. Também podem resgatar o FGTS os trabalhadores que tiverem dependentes nessas condições (cônjuge, filhos, irmãos menores de 21 anos ou inválidos e pais)
Saque do Programa de Integração Social (PIS) e do Programa de Formação do Patrimônio do Servidor Público (Pasep)	Antes de 1988, o PIS/Pasep era depositado em uma conta vinculada ao trabalhador. O PIS diz respeito aos empregados atuantes no setor privado e tem o pagamento sob responsabilidade da Caixa Econômica Federal, enquanto o Pasep beneficia funcionários do setor público e é pago pelo Banco do Brasil. Só poderá fazer o saque aquele trabalhador cadastrado como participante do Fundo PIS/Pasep até 4 de outubro de 1988 que ainda não sacou. Os pacientes com câncer e/ou trabalhadores que tiverem dependentes nessas condições (cônjuges, filhos, irmãos menores de 21 anos ou inválidos, e pais previamente registrados no INSS ou no imposto de renda) podem sacar.
Passe livre municipal	Cada município define suas normas. Em Juiz de Fora (Minas Gerais), por exemplo, todos os pacientes com câncer que estejam em período de tratamento têm direito a esse benefício. Caso o paciente necessite de um acompanhante, ele também terá direito ao transporte municipal, desde que a necessidade seja indicada em laudo médico. Além disso, o estatuto da pessoa idosa, *Lei nº 10.741*, de 1º de outubro de 2003, prevê em seu artigo 39 que idosos com mais de 65 anos têm direito à gratuidade para utilizar os transportes públicos coletivos, exceto nos serviços especiais. Para que o idoso tenha acesso à gratuidade, ele deve apresentar qualquer documento que comprove sua idade.
Passe livre interestadual	Transporte coletivo interestadual por ônibus, trem ou barco, incluindo transporte interestadual semiurbano, sem direito à gratuidade para acompanhante. Caso o paciente necessite de acompanhante, ele também terá direito ao transporte, desde que a necessidade seja indicada em laudo médico. É emitido pelo Governo Federal. Os portadores de deficiência física, mental, auditiva ou visual, com renda mensal *per capita* de até um salário mínimo, têm direito a esse benefício.
Isenção de Imposto Predial e Territorial Urbano (IPTU)	É um tributo cobrado sobre a posse de todo tipo de imóvel localizado em zona urbana. Não há lei nacional que garanta a isenção; é importante conhecer a legislação do município onde o paciente reside. Em Juiz de Fora, por exemplo, o paciente deve fazer o cadastro no portal eletrônico da Procuradoria da Prefeitura, preencher o formulário, anexar os documentos necessários e aguardar análise do órgão responsável.
Tratamento Fora de Domicílio (TFD) no Sistema Único de Saúde (SUS)	Tem por objetivo garantir o acesso de pacientes moradores de um município a serviços assistenciais em outro município, ou ainda de um estado para outro. Envolve a garantia de transporte, hospedagem e ajuda de custo para alimentação. É concedido exclusivamente aos pacientes atendidos no SUS.
Isenção do imposto de renda na aposentadoria, pensão e reforma	Imposto de renda é um tributo cobrado pelo governo sobre o salário de trabalhadores, atividades econômicas e rendimentos. A declaração é anual. De acordo com a *Lei nº 7.713*, de 22 de dezembro de 1988, a pessoa com câncer está isenta do imposto de renda relativo aos rendimentos de aposentadoria, reforma e pensão, inclusive as complementações recebidas de entidade privada e pensão alimentícia. Em 2022, por exemplo, também não precisou declarar imposto de renda quem recebeu rendimentos tributáveis cuja soma anual foi menor que R$ 28.559,70 (e.g., equivalente a um rendimento mensal menor que R$ 2.380,00).
Prioridade na tramitação de processos	De acordo com a *Lei Federal nº 12.008*, de 29 de julho de 2009, o paciente com câncer tem prioridade na tramitação de processos judiciais e administrativos.

Fonte: *Lei nº 14.238/2021* (Brasil ^5^); Instituto Nacional de Câncer ^6^; Oncoguia ^7^; *Portaria nº 55/1999* (Secretaria de Atenção à Saúde, Ministério da Saúde ^8^).

Há poucos estudos relacionados ao conhecimento do paciente portador de câncer sobre seus direitos sociais ^9^^,^^10^^,^^11^^,^^12^. Avaliar esse aspecto, principalmente entre aqueles atendidos no SUS, é fundamental para assegurar o compromisso de prover condições econômicas de acesso a alimentação, habitação, educação, meio ambiente, trabalho, transporte e lazer, fortalecendo, assim, os cuidados integrais ao usuário ^13^^,^^14^. O objetivo deste estudo foi, portanto, analisar a proporção de indivíduos em tratamento oncológico de um centro de referência do SUS que referiram conhecer os direitos específicos pesquisados.

## Metodologia

Trata-se de um estudo observacional descritivo. A pesquisa foi planejada e executada pelo serviço social do Hospital Associação Feminina de Prevenção e Combate ao Câncer de Juiz de Fora (ASCOMCER), Minas Gerais, Brasil, onde o tratamento é primordialmente direcionado a pacientes do SUS (94%). O critério de inclusão para o estudo foi definido como todos os pacientes oncológicos maiores de idade que agendaram consulta para serem tratados pela primeira vez nos ambulatórios de radioterapia e quimioterapia do Hospital ASCOMCER entre março e julho de 2022. Aqueles casos com recidiva de câncer, casos de segundo (ou mais) câncer e/ou pacientes sem condições clínicas para participar da pesquisa foram excluídos do estudo.

Em razão dos dados do registro hospitalar de câncer sobre os casos novos analíticos em que os pacientes realizaram algum tipo de tratamento específico de controle de neoplasias malignas no Hospital ASCOMCER (cerca de 500 em 2020) ^15^, estimou-se entrevistar pelo menos 200 pacientes em cinco meses. Para os pacientes que foram abordados no dia da consulta para iniciar o tratamento e concordaram em participar do estudo (n = 62), foi realizada uma entrevista individual por meio de um questionário envolvendo perguntas destinadas a coletar informações sociodemográficas, de perfil de elegibilidade para os direitos selecionados e de conhecimento dos direitos específicos (conforme Material Suplementar 1: https://cadernos.ensp.fiocruz.br/static//arquivo/suppl-e00096023-1_1023.pdf; e Material Suplementar 2: https://cadernos.ensp.fiocruz.br/static//arquivo/suppl-e00096023-2_8377.pdf). 

O desfecho principal do estudo, ou seja, o conhecimento sobre cada direito selecionado, foi avaliado a partir de duas questões:

(1) uma pergunta sobre conhecimento espontâneo: O(A) Sr(a) conhece algum direito do paciente oncológico? Se a resposta for afirmativa, qual(is)?;

(2) uma pergunta sobre conhecimento direcionado: O(A) Sr(a) sabe se o paciente oncológico tem direito a [direito específico baseado no Quadro 1]? Com opções de resposta (a) não; (b) sim, sempre; (c) sim, sob determinados requisitos; e (d) não conheço esse benefício. As opções (b) e (c) foram agrupadas para definir a resposta afirmativa.

Para o conjunto de todos os pacientes, foi calculada a proporção de “conhecimento de pelo menos algum dos direitos selecionados”, baseada na pergunta de conhecimento espontâneo ou na de conhecimento direcionado.

Calcularam-se as proporções de pacientes elegíveis para solicitar cada direito selecionado e, posteriormente, as proporções de conhecimento espontâneo ou direcionado específico por direito, adotando como denominador a população elegível (ou a não elegível) para solicitar o direito. A elegibilidade para poder solicitar determinado direito foi definida a partir das exigências estabelecidas em lei de acordo com a condição socioeconômica e/ou de saúde em que o paciente estivesse no momento da entrevista (conforme Material Suplementar 1: https://cadernos.ensp.fiocruz.br/static//arquivo/suppl-e00096023-1_1023.pdf; e Material Suplementar 2: https://cadernos.ensp.fiocruz.br/static//arquivo/suppl-e00096023-2_8377.pdf).

Para verificar a associação entre a proporção de conhecimento do direito selecionado e o *status* de elegibilidade para solicitá-lo, foi utilizado o teste qui-quadrado de Pearson; quando do não atendimento dos pressupostos para seu uso, foi utilizado o teste exato de Fisher.

Todas as análises foram realizadas com o programa estatístico Stata, versão 15.0 (https://www.stata.com).

O estudo foi aprovado pelo Comitê de Ética em Pesquisa do Instituto Nacional de Câncer José Alencar Gomes da Silva (CEP-Inca; CAAE 53368221.50000.5274) no dia 25 de fevereiro de 2022.

## Resultados

No total, 88 pacientes chegaram para iniciar pela primeira vez o tratamento no Hospital ASCOMCER. Destes, cerca 26,1% não apresentaram condições clínicas para serem entrevistados e 3,4% se recusaram a assinar o consentimento informado e foram excluídos do estudo. Dos 62 pacientes entrevistados, predominaram mulheres (51,6%), indivíduos com idade superior a 64 anos (54,8%), com nível educacional abaixo do Ensino Médio (66,1%), vivendo em domicílios com renda *per capita* de no máximo um salário mínimo (75%), aposentados (46,8%), atendidos pelo SUS (91,9%) e provenientes da cidade de Juiz de Fora (58,1%). Predominaram, ainda, pacientes com câncer de mama (21%) ou de próstata (21%) e em estadiamento III (32,3%) (dados não mostrados em tabela).

Entre todos os pacientes, a proporção de “conhecimento de pelo menos algum dos direitos selecionados” baseada na pergunta de conhecimento espontâneo (21%) foi menor quando comparada à respectiva proporção fundamentada na pergunta de conhecimento direcionado (62,9%) (Tabela 1). Nota-se que nove dos 15 direitos selecionados exibiram proporção de pacientes elegíveis superior a 10%, variando de 17,7% para “saque do FGTS” a 100% para “prioridade na tramitação de processos”. Desses nove direitos, aqueles que apresentaram pelo menos 1/3 de pacientes elegíveis com “conhecimento direcionado” foram apenas “auxílio-doença” e “passe livre municipal”, com proporções estatisticamente superiores às encontradas entre os pacientes não elegíveis para solicitar esses direitos. A proporção de conhecimento do direito “aposentadoria por invalidez” foi bem inferior à proporção de conhecimento do direito “auxílio-doença” entre os mesmos pacientes elegíveis (70,6% *vs.* 11,8%). A alta proporção de pacientes elegíveis para os direitos “isenção do imposto de renda” (59,7%), “isenção de imposto sobre propriedade predial e territorial urbana” (80,7%) e “prioridade na tramitação de processos” (100%) foi acompanhada de baixíssima proporção de conhecimento sobre eles (2,7%, 6% e 3,2%, respectivamente). Finalmente, vale a pena destacar a ausência total de conhecimento sobre o direito ao “saque do Programa de Integração Social (PIS) ou do Programa de Formação do Patrimônio do Servidor Público (Pasep)” entre os quase 20% de pacientes elegíveis para ele.

**Tabela 1:** Proporção de conhecimento de direitos selecionados, segundo tipo de pergunta e *status* de elegibilidade ao direito. Hospital da Associação Feminina de Prevenção e Combate ao Câncer de Juíz de Fora, Minas Gerais, Brasil, 2022.

Direitos selecionados	População (n = 62)	Conhecimento: pergunta espontânea			Conhecimento: pergunta direcionada		
	Elegíveis	Não elegíveis	Elegíveis	Total	Não elegíveis	Elegíveis	Total
	%	%	%	%	%	%	%
Algum direito	n.a.	n.a.	n.a.	21,0	n.a.	n.a.	62,9
LOAS	0,0	4,8	n.a.	4,8	11,3	n.a.	11,3
Auxílio-doença	27,4	8,9	29,4	14,5	28,9	70,6 *	40,3
Invalidez	27,4	4,4	5,9	4,8	24,5	11,8	21,0
IPI	6,5	3,4	0,0	3,2	5,2	0,0	4,8
IPVA	1,6	1,6	0,0	1,6	6,6	0,0	6,4
ICMS	6,5	1,7	0,0	1,6	1,7	0,0	1,6
SFH	1,6	1,7	0,0	1,6	3,2	0,0	3,2
FGTS	17,7	1,9	9,1	3,2	7,8	18,2	9,7
PIS/Pasep	19,4	2,0	0,0	1,6	4,0	0,0	3,2
Passe municipal	59,7	4,0	5,5	4,8	8,0	40,5 *	27,4
Passe interestadual	6,5	1,7	0,0	1,6	5,2	25,0	6,5
IPTU	80,7	0,0	2,0	1,6	0,0	6,0	4,8
TFD	91,9	0,0	7,0	6,5	20,0	28,1	27,4
IRPF	59,7	0,0	2,7	1,6	8,0	2,7	4,8
Processos	100,0	0,0	0,0	0,0	0,0	3,2	3,2

FGTS: Fundo de Garantia por Tempo de Serviço (saque); ICMS: Imposto sobre Circulação de Mercadorias e Serviços (isenção); Invalidez: aposentadoria por invalidez; IPI: Imposto sobre Produtos Industrializados (isenção); IPVA: Imposto sobre a Propriedade de Veículos Automotores (isenção para veículos adaptados); IPTU: Imposto Predial e Territorial Urbano (isenção); IRPF: Imposto sobre a Renda das Pessoas Físicas (isenção do imposto na aposentadoria, pensão e reforma); LOAS: Lei Orgânica de Assistência Social; n.a.: não se aplica; PIS/Pasep: Programa de Integração Social ou do Programa de Formação do Patrimônio do Servidor Público (saque); Processos: prioridade na tramitação de processos; SFH: Sistema Financeiro de Habitação em caso de invalidez ou morte (quitação de financiamento de imóvel); TFD: tratamento fora de domicílio.

* Valor de p < 0,05 (teste exato de Fisher).

## Discussão

A proporção de conhecimento dos direitos específicos por meio de perguntas direcionadas entre todos os pacientes oncológicos atendidos no Hospital ASCOMCER (elegíveis ou não para os direitos) esteve sempre abaixo de 40% (com exceção do “auxílio-doença”), sendo que, para cerca de 70% dos direitos selecionados, essa proporção ficou abaixo de 10%. Ao analisar a proporção de pacientes que “conheciam pelo menos algum dos direitos selecionados”, o percentual encontrado foi de 62,9%.

Estudo realizado em 2008 em uma unidade de tratamento oncológico para pacientes com perfil socioeconômico mais elevado do que o dos atendidos no Hospital ASCOMCER encontrou percentual relativamente parecido (55%) ^10^. É importante assinalar, contudo, que a proporção de conhecimento de direitos específicos não foi a mesma entre os estudos, o que pode, de certa forma, refletir diferenças na contribuição relativa da proporção de pacientes elegíveis para cada direito analisado. Outro estudo realizado em 2015 em uma unidade pediátrica oncológica encontrou elevada proporção de conhecimento do Benefício de Prestação Continuada (BPC)/Lei Orgânica de Assistência Social (LOAS) ^9^, mas 50% dos pacientes que o solicitaram tiveram o pedido negado, o que pode sugerir, entre outros aspectos, a limitação deste estudo de não ter avaliado a proporção de conhecimento dos direitos específicos em função da elegibilidade dos pacientes para eles. O estudo realizado junto aos pacientes do Hospital ASCOMCER tem o ineditismo, portanto, de avaliar a proporção de conhecimento por direito selecionado em uma população para a qual esse direito tinha evidentemente mais relevância (ou seja, na população elegível para solicitá-lo).

Aproximadamente 1/3 dos direitos selecionados apresentaram uma “relação desequilibrada” entre a proporção de pacientes elegíveis (> 50%) e a proporção de conhecimento do direito entre a população elegível (< 50%). Considerando que a maioria das pessoas atendidas para tratamento pela primeira vez no Hospital ASCOMCER tinha condição socioeconômica precária, o impacto populacional obtido com a disseminação desse tipo de informação em pacientes com câncer atendidos pelo SUS pode ser, portanto, relevante para atenuar as desigualdades sociais do país. Nesse ponto, o profissional de saúde, sobretudo o assistente social do Hospital ASCOMCER (ou de qualquer outra instituição), é fundamental, tendo em vista que ele atua como facilitador para que o paciente e seus familiares tenham acesso às informações sobre seus direitos e saibam como solicitá-los ^9^^,^^10^^,^^11^^,^^12^^,^^13^^,^^14^. O contato com pacientes e familiares é, de fato, fundamental, pois muitas vezes eles acabam expressando ao assistente social suas dúvidas, queixas e preocupações acerca do momento que estão vivendo. Com isso, cabe ao assistente social acolher e compreender as solicitações apresentadas pelos indivíduos ^13^.

É interessante notar que, no caso do “passe livre municipal” e do “tratamento fora de domicílio”, provavelmente, muitos dos pacientes elegíveis que já usufruem desses direitos nem percebem que eles somente são concedidos sob determinadas condições às quais os pacientes fazem jus. De fato, quando vemos a enorme diferença entre a resposta à pergunta de conhecimento espontâneo e a resposta à pergunta de conhecimento direcionado fica ainda mais evidente a necessidade de reforçar para essa população que uma parte dela já está, provavelmente, sendo beneficiada. Além dos benefícios econômicos advindos do direito, a consciência de ter acesso a algum direito garantido em lei em razão da sua condição de saúde pode reforçar sentimentos importantes de pertencimento social, os quais auxiliam na jornada do tratamento ^16^^,^^17^.

Infelizmente, a quantidade de pacientes atendidos pela primeira vez no Hospital ASCOMCER entre os meses de março e julho de 2022 ficou bem aquém daquela inicialmente prevista, o que acabou prejudicando a significância estatística de algumas comparações realizadas. Houve, de fato, perda diferencial de quase 30% dos potenciais pacientes elegíveis para este estudo, muito em razão do agravamento das condições clínicas daqueles que provavelmente retardaram o início do tratamento devido à pandemia de COVID-19 ^15^. Outra limitação inerente ao estudo é o fato de que algumas informações utilizadas para estabelecer o critério de elegibilidade para solicitar determinado direito foram autorreferidas no momento da aplicação do questionário.

## Conclusão

A proporção de conhecimento dos pacientes acerca dos direitos para os quais eles eram elegíveis ficou abaixo de 10% para mais da metade dos direitos pesquisados. É fundamental orientar os pacientes quanto aos seus direitos e benefícios, de forma a auxiliar na identificação de recursos que favoreçam o processo de tratamento da doença.
